# ColdZyme Maintains Integrity in SARS-CoV-2-Infected Airway Epithelia

**DOI:** 10.1128/mBio.00904-21

**Published:** 2021-04-27

**Authors:** W. Posch, J. Vosper, V. Zaderer, A. Noureen, S. Constant, R. Bellmann-Weiler, C. Lass-Flörl, D. Wilflingseder

**Affiliations:** a Institute of Hygiene and Medical Microbiology, Medical University of Innsbruck, Innsbruck, Austria; b Institute of Medical Biochemistry, Geneva, Switzerland; c Epithelix Sàrl, Geneva, Switzerland; d University Hospital of Internal Medicine II, Medical University of Innsbruck, Innsbruck, Austria; Max Planck Institute for Infection Biology

**Keywords:** ColdZyme, SARS-CoV-2, airway epithelia, anaphylatoxins, antiviral response

## Abstract

SARS-CoV-2 infection causing the COVID-19 pandemic calls for immediate interventions to avoid viral transmission, disease progression, and subsequent excessive inflammation and tissue destruction. Primary normal human bronchial epithelial cells are among the first targets of SARS-CoV-2 infection. Here, we show that ColdZyme medical device mouth spray efficiently protected against virus entry, excessive inflammation, and tissue damage. Applying ColdZyme to fully differentiated, polarized human epithelium cultured at an air-liquid interphase (ALI) completely blocked binding of SARS-CoV-2 and increased local complement activation mediated by the virus as well as productive infection of the tissue model. While SARS-CoV-2 infection resulted in exaggerated intracellular complement activation immediately following infection and a drop in transepithelial resistance, these parameters were bypassed by single pretreatment of the tissues with ColdZyme mouth spray. Crucially, our study highlights the importance of testing already evaluated and safe drugs such as ColdZyme mouth spray for maintaining epithelial integrity and hindering SARS-CoV-2 entry within standardized three-dimensional (3D) *in vitro* models mimicking the *in vivo* human airway epithelium.

## Abstract (precis)

Although our understanding of COVID-19 continuously progresses, essential questions regarding prophylaxis and treatment remain open. A hallmark of severe SARS-CoV-2 infection is a hitherto-undescribed mechanism leading to excessive inflammation and tissue destruction associated with enhanced pathogenicity and mortality.

## INTRODUCTION

The emergence in late 2019 of COVID-19 (coronavirus disease 19) caused by the novel SARS-CoV-2 has given rise to an unprecedented global health crisis. As of mid-March 2021, more than 119 million SARS-CoV-2 cases have been confirmed with approximately 2.6 million deaths worldwide (https://covid19.who.int/). Similarly to SARS-CoV or human coronavirus NL63 (HCoV-NL63), SARS-CoV-2 enters the cell via high-affinity interaction of the structural spike (S) glycoprotein with the angiotensin-converting enzyme 2 (ACE2) receptor (1–3). ACE2 is ubiquitously expressed in nasal and respiratory epithelium, lung, heart, kidney, and intestine but is rarely found on immune cells (4, 5). Due to high ACE2 expression in nose and mouth and due to a 10- to 20-fold-higher ACE2-binding affinity of the receptor binding domain (RBD) in the S1 unit of the S protein compared to SARS-CoV (4), SARS-CoV-2 is highly contagious and rapidly spreads within populations, if social distancing measures are not adhered to. Up-to-date efficient treatments to avoid the transfer of the virus from one to another are lacking (1).

ColdZyme is a class III medical device (CE-marked) composed of glycerol, water, buffer, CaCl_2_, menthol, and trypsin from the Atlantic cod (Gadus morhua) (6) (ClinicalTrials.gov, ID: NCT03901846). The ColdZyme mouth spray forms a physical barrier that interferes with entry of common cold viruses, which subsequently become trapped and inactivated (7, 8). Therefore, it is thought that ColdZyme prevents entry of viruses into host cells. *In vitro* and *in vivo* analyses revealed that ColdZyme inactivated viruses such as rhinovirus, respiratory syncytial virus (RSV), or influenza virus in a range between 60% and 100%, when applied for 20 min at 35 to 37°C (7). The mouth spray exerted a higher inactivation capacity for the enveloped viruses RSV and influenza virus than for nonenveloped rhinoviruses. The oral mucosa itself is very well protected against proteolytic enzymes such as trypsin, an ingredient of ColdZyme, by protease inhibitors and mucins, and cod trypsin is more sensitive to pH and heat, thereby explaining the high safety profile of the ColdZyme mouth spray (7). Gudmundsdottir et al. recently illustrated that the ColdZyme mouth spray also efficiently inactivated SARS-CoV-2 and HCoV-229E *in vitro* in Vero E6 and MRC-5 cells (8). Since early events occurring directly after SARS-CoV-2 transmission to respiratory tissues can influence the outcome in the context of disease severity, here we evaluated the protective properties of the ColdZyme mouth spray in more detail. In some patients, infection with COVID-19 results in overshooting activation of the immune response at epithelial barriers and the generation of a proinflammatory milieu (9, 10). These excessive immune responses triggered by incoming viruses result in extensive tissue destruction during severe cases, resulting in tissue injury and multiorgan failure (11, 12). Very recently, Ramlall et al. identified, in addition to type I IFN- and IL-6-dependent inflammatory responses, a robust engagement of complement and coagulation pathways following SARS-CoV-2 infection (13). In our study, we applied the ColdZyme mouth spray to highly differentiated primary nasal and bronchial epithelial three-dimensional (3D) tissue models prior to *in vitro* infection using SARS-CoV-2-clinical strains (14, 15). While we detected rapid tissue destruction with concomitant innate immune (complement C3) activation upon SARS-CoV-2 exposure and in infected cultures, the ColdZyme mouth spray completely restored tissue integrity and significantly downmodulated local complement production and in association thereby returned C3a levels to normal. Our results point toward an easy-to-use, novel therapeutic intervention strategy whereby ColdZyme mouth spray is administered in order to prevent an excessive inflammatory response and its associated pathological consequences.

## RESULTS

### ColdZyme mouth spray protects from SARS-CoV-2 attachment and intracellular (IC) C3 activation in primary human bronchial epithelial (NHBE) cells.

In first experiments, we monitored SARS-CoV-2 attachment to primary normal human bronchial epithelial (NHBE) cells in the absence and presence of the ColdZyme mouth spray. For binding and infection assays of these cells, a multiplicity of infection (MOI) of 0.1 was used, which is consistent with infection by other coronaviruses, such as HCoV-NL63, SARS-CoV, or Middle East respiratory syndrome (MERS)-CoV and as used in other settings by Zhu et al. to determine morphogenesis and cytopathic effects of SARS-CoV-2 infection in human airway epithelial (HAE) cells (16–19).

**FIG 1:**
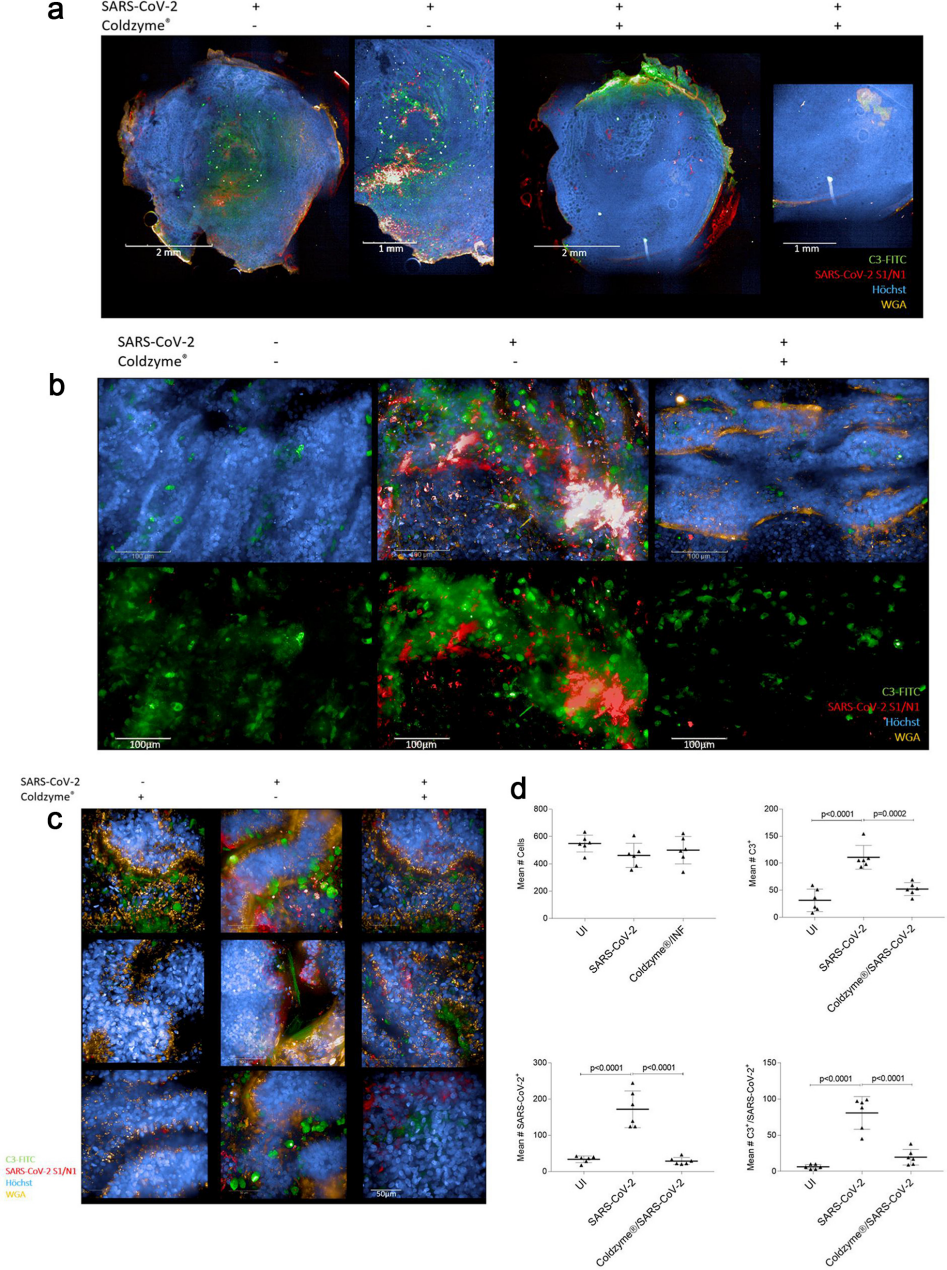
ColdZyme mouth spray protects primary human airway epithelial (HAE) cells from SARS-CoV-2 binding and innate immune activation. Visualization of virus binding (SARS-CoV-2-S1/N, red) and complement (C3-FITC, green) in SARS-CoV-2-infected 3D pseudostratified epithelia. Multilayered epithelia were apically treated with solvent control or ColdZyme mouth spray prior to exposure to SARS-CoV-2 (MOI 0.1). After 2 h, filters were fixed; stained for Hoechst (blue), SARS-CoV-2-S1/N (red), complement C3 (green), and WGA (orange); and then analyzed by HCS. (a) Overview on the whole Transwell filter of solvent (left)- and ColdZyme (right)-pretreated and infected HAE cultures using ×5 magnification. One representative filter and one detail out of the filter are illustrated. Scale bars represent 2 mm or 1 mm as indicated. (b) Z-stacks of six fields of uninfected (UI, left), SARS-CoV-2-exposed (middle), and ColdZyme/SARS-CoV-2 (right)-exposed cells were analyzed using the Operetta CLS HCS and the 63× water objective. Cells were stained using C3-FITC (green) as indicator for innate immune activation, SARS-CoV-2-S1/N-Alexa 594 (red) for virus detection, Hoechst stain for imaging nuclei (blue), and WGA for staining lectins (orange). Massive IC C3 mobilization and SARS-CoV-2 binding/uptake were monitored in SARS-CoV-2-exposed cultures (middle), while no virus and low C3 signals were detected in UI (left) and ColdZyme/SARS-CoV-2-exposed (right) cultures. Scale bars represent 100 μm, and three independent experiments were performed. (c) Z-stacks of several representative single fields of SARS-CoV-2-exposed regions under the different conditions (ColdZyme/UI, left; SARS-CoV-2, middle; ColdZyme/SARS-CoV-2, right) are shown. Scale bars represent 50 μm, and three independent experiments were performed. (d) More than 2,500 cells (upper left) were analyzed for their expression of C3 (upper right) and SARS-CoV-2 (lower left), where up to 50% of the analyzed SARS-CoV-2-infected cells were stained positive for C3 (upper right) or virus (lower left), while only background signals were detected in UI or ColdZyme/SARS-CoV-2-exposed cells. Too, significantly higher levels of SARS-CoV-2/C3 double-positive cells were analyzed in the infected cultures compared to UI or treated ones (lower right). Statistical significances were analyzed on >2,500 cells with GraphPad Prism software using one-way ANOVA and Tukey’s posttest.

One hub of ColdZyme or solvent control was carefully sprayed from about 2.5- to 3-cm distance onto the apical side of fully differentiated, pseudostratified epithelia cultured at air-liquid interphase (ALI) (14, 15) to realistically mimic the distribution within the oral or nasal cavity. ColdZyme or solvent was preincubated for 30 min prior to applying a clinical isolate derived from a SARS-CoV-2 patient for 90 min. The clinical specimen was anonymized before use, and the study has been approved by the ethics committee of the Medical University of Innsbruck (approval no. ECS1166/2020). Uninfected tissues treated with either solvent (UI) or ColdZyme mouth spray alone (ColdZyme/UI) served as controls. After the incubation period, the tissue models were fixed and stained for immunofluorescence (IF) analyses using Alexa 594-labeled antibodies against the SARS-CoV-2 spike 1 (S1) and nucleocapsid (N) proteins to detect virus binding, Hoechst stain for nuclei, wheat germ agglutinin (WGA) for glycocalyx, and complement component C3 as marker for innate immune activation of NHBE cells. Here, intracellular (IC) C3 was used as an indicator for tissue damage during SARS-CoV-2 infection of NHBE cells, since we recently found that infection in primary airway epithelial cells was accompanied by extensive induction of IC C3 and secretion of the anaphylatoxin C3a from HAE cells (20). NHBE tissue models illustrated an excessive binding and uptake of SARS-CoV-2 (red), already detectable when imaging the overview of the whole Transwell filter containing the fully differentiated epithelium, going along with concomitant activation of IC C3 (green) (Fig. 1a, left two panels). In contrast, nearly no virus (red) or IC C3 (green) signal was observed when the epithelium was pretreated with ColdZyme mouth spray (Fig. 1a, right two panels). In these experiments, red and green signals from the directly labeled antibodies were solely found at the edges of the filter membrane, indicating an efficient mucociliary clearance (Fig. 1a, right two panels).

Upon exposure of epithelia to SARS-CoV-2 for 90 min, massive IC C3 mobilization and tissue disruption were monitored immediately thereafter as illustrated in Fig. 1b and c, middle panels. In contrast, not only was binding/uptake of SARS-CoV-2 hampered, if tissues were pretreated with the ColdZyme mouth spray, but also the epithelia were rescued from IC C3 mobilization and tissue damage (Fig. 1b and c, right panel). IC C3 levels were comparable to those of buffer-treated (Fig. 1b, left) or ColdZyme-treated, uninfected samples (Fig. 1c, left). Next, we analyzed and quantified signals from similar amounts (∼2,500 cells analyzed, Fig. 1d, upper left) of cells in UI, SARS-CoV-2-exposed, and ColdZyme/SARS-CoV-2-exposed, pseudostratified respiratory epithelia. We found highly significant differences in numbers of C3^+^ (Fig. 1d, upper right), SARS-CoV-2^+^ (Fig. 1d, lower left), and C3/SARS-CoV-2 double-positive (Fig. 1d, lower right) cells between SARS-CoV-2-infected cultures and uninfected and ColdZyme-treated/SARS-CoV-2-infected, primary respiratory cultures. These analyses demonstrate that the immediate tissue destruction and intracellular C3 mobilization induced in NHBE cultures upon SARS-CoV-2 interactions can be avoided by pretreatment of epithelia with ColdZyme mouth spray.

### ColdZyme mouth spray maintains epithelial integrity and dampens innate immune activation upon SARS-CoV-2 infection of NHBE cultures.

**FIG 2:**
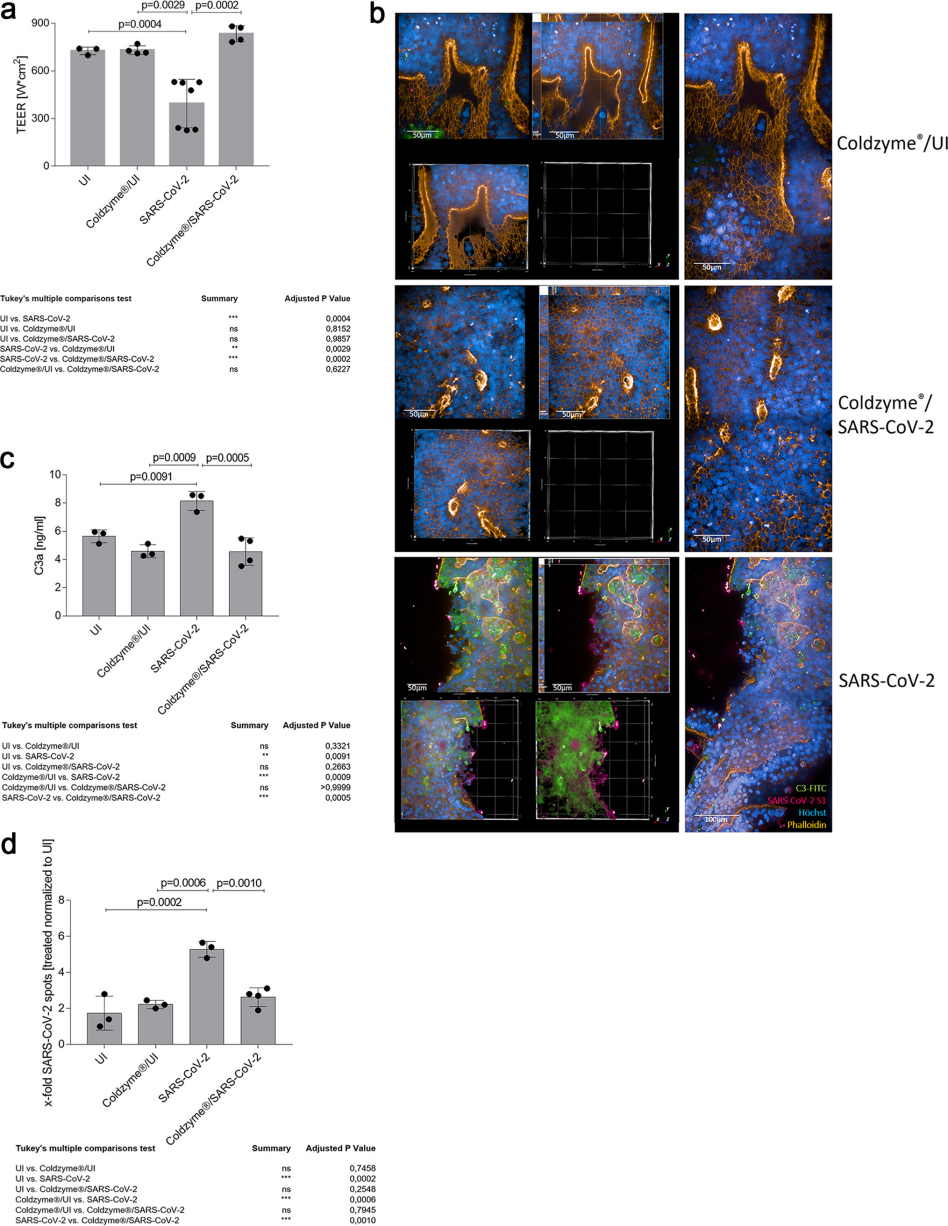
Disruption of epithelial integrity is contingent on extensive C3 mobilization and C3a secretion by SARS-CoV-2 and can be avoided by ColdZyme pretreatment. (a) Multilayered epithelia were infected by apical addition of SARS-CoV-2 (MOI 0.1) with or without ColdZyme pretreatment and incubated for 72 h. TEER was measured using an EVOM voltohmmeter. TEER in Ω/cm^2^ was determined for all conditions (UI, ColdZyme/UI, SARS-CoV-2, ColdZyme/SARS-CoV-2) and plotted on a bar graph. Bars represent the mean + SD from 3 to 6 independent pseudostratified epithelia. Statistical significance was calculated using one-way ANOVA with Tukey’s multiple-comparison test. (b) Image analyses of ColdZyme/UI, ColdZyme/SARS-CoV-2, and SARS-CoV-2 primary HAE cells. Filters were differentially treated according to the labeling, fixed after 3 days, and stained using C3-FITC (green), SARS-CoV-2-S1 (red), phalloidin (orange), and Hoechst stain (blue). Image analyses revealed intact epithelial tissue structures (orange) and nuclei (blue) in ColdZyme/UI (upper panel) and ColdZyme/SARS-CoV-2-infected (middle panel) HAE, while tissue integrity was disrupted in SARS-CoV-2-infected cultures, which also illustrated high C3 and SARS-CoV-2-S1 staining (red and green, lower panel). Under each condition, one representative Z-stack (upper left), xyz stack (upper middle), 3D analyses of all stainings (lower left) and virus/C3 stainings (lower middle), and two imaged fields (right) are depicted. Infection experiments were performed three times independently. Scale bars represent 50 μm or 100 μm as indicated. (c) C3a level determination in SARS-CoV-2-infected or ColdZyme-pretreated/SARS-CoV-2-infected pseudostratified epithelia. Multilayered epithelia were pretreated or not with ColdZyme prior to infection with SARS-CoV-2 (MOI 0.1) for 72 h. Uninfected (UI) and ColdZyme/UI cultures served as controls. Basolateral supernatants were harvested, and C3a levels were determined using a BD OptEIA human C3a ELISA kit. C3a levels in ng/ml were determined for all UI and infected epithelia and plotted on a bar graph. Dots represent values from independent experiments. Statistical significance was calculated using one-way ANOVA with Tukey’s multiple-comparison test. (d) Spot analyses were performed on UI, ColdZyme/UI, SARS-CoV-2-infected, and ColdZyme/SARS-CoV-2-infected HAE cultures. SARS-CoV-2 spots (Alexa 594) were counted on an average of 1,200 cells (Hoechst, nuclear count; Alexa 647, cytoplasm) using the RMS spot analyses (Harmony software; Operetta CLS, Perkin-Elmer). Due to background spots in the UI and ColdZyme/UI cultures probably due to autofluorescence of dead cells in the 3D cultures, all conditions were normalized to UI. One out of three representative experiments was set as 1.0, and all other samples were normalized to this. The experiment was repeated at least 3 times, and one-way ANOVA with Tukey’s multiple-comparison test was used to calculate statistical significance. ns, not significant.

To monitor if ColdZyme mouth spray is also effective against SARS-CoV-2 infection over the long term, NHBE cultures were treated as indicated above but kept in culture for another 3 days. At day 3 postinfection (d3pI), whole-tissue cultures were first analyzed for transepithelial electrical resistance (TEER) (Fig. 2a), an indicator for tissue integrity. These analyses revealed that upon SARS-CoV-2 infection (SARS-CoV-2), TEER values significantly dropped compared to buffer (UI)- or ColdZyme-treated uninfected controls (ColdZyme/UI) (Fig. 2a). While in UI or ColdZyme/UI tissue culture TEER values ranged from 700 to 780 Ω/cm^2^, this range dropped to 250 to 510 Ω/cm^2^ in SARS-CoV-2-infected cells (SARS-CoV-2) (Fig. 2a, left). Tissue integrity was completely rescued in infection experiments also on d3pI, if ColdZyme was applied prior to SARS-CoV-2 exposure, and TEER values were 825 to 840 Ω/cm^2^ (Fig. 2a; ColdZyme/SARS-CoV-2). TEER values in these cultures were significantly higher than in SARS-CoV-2-infected cultures and were even higher than those under UI or ColdZyme/UI conditions (Fig. 2a). TEER analyses are in accord with IF analyses performed using various markers (SARS-CoV-2 N/S1 [Fig. 2b; red], C3 [Fig. 2b; green], phalloidin to detect F-actin [Fig. 2b; orange], Hoechst stain [Fig. 2b; blue]). The staining revealed a significant infection of the tissue models with SARS-CoV-2 with high IC C3 production and strong tissue disruption (Fig. 2b, SARS-CoV-2). 3D and xyz analyses of the two signals not only revealed a superficial localization of SARS-CoV-2 and C3 but also showed that the infection and intracellular complement mobilization penetrated deep into the tissue layers (Fig. 2b, SARS-CoV-2). In contrast, no signals for SARS-CoV-2 and C3 were apparent in the ColdZyme-treated, UI control and ColdZyme-treated, SARS-CoV-2-infected HAE cultures (Fig. 2b, ColdZyme/UI and ColdZyme/SARS-CoV-2). Avoidance of innate immune activation by pretreatment with the mouth spray was further confirmed by analyzing the anaphylatoxin C3a in culture subnatants, taken on d3pI from the basolateral side of the cultures (Fig. 2c). In accord with TEER and image analyses, these experiments, too, revealed a significantly higher C3a secretion from SARS-CoV-2-infected cultures than from UI, ColdZyme/UI, and ColdZyme/SARS-CoV-2 cultures (Fig. 2c). Finally, viral loads in differentially treated tissues were determined by viral spot analyses of at least 1,200 cells. Lowest background fluorescent signals of UI controls from one independent experiment (400 cells) were set as 1 to normalize all other conditions. While SARS-CoV-2-infected cultures illustrated significantly higher virus spots (5.8-fold) compared to UI and ColdZyme/UI cultures, viral spot analyses from ColdZyme-pretreated and SARS-CoV-2-infected cultures gave results as low as those of UI controls and significantly lower than those of SARS-CoV-2-infected cultures (Fig. 2d). Here, we found that a single application of ColdZyme mouth spray blocked SARS-CoV-2 infection of HAE cultures in terms of rescuing tissue integrity, significantly reducing viral loads, and inhibiting innate immune activation.

### ColdZyme mouth spray inhibits SARS-CoV-2 infection in highly differentiated nasal epithelial cultures (MucilAir) despite IC C3 mobilization.

**FIG 3:**
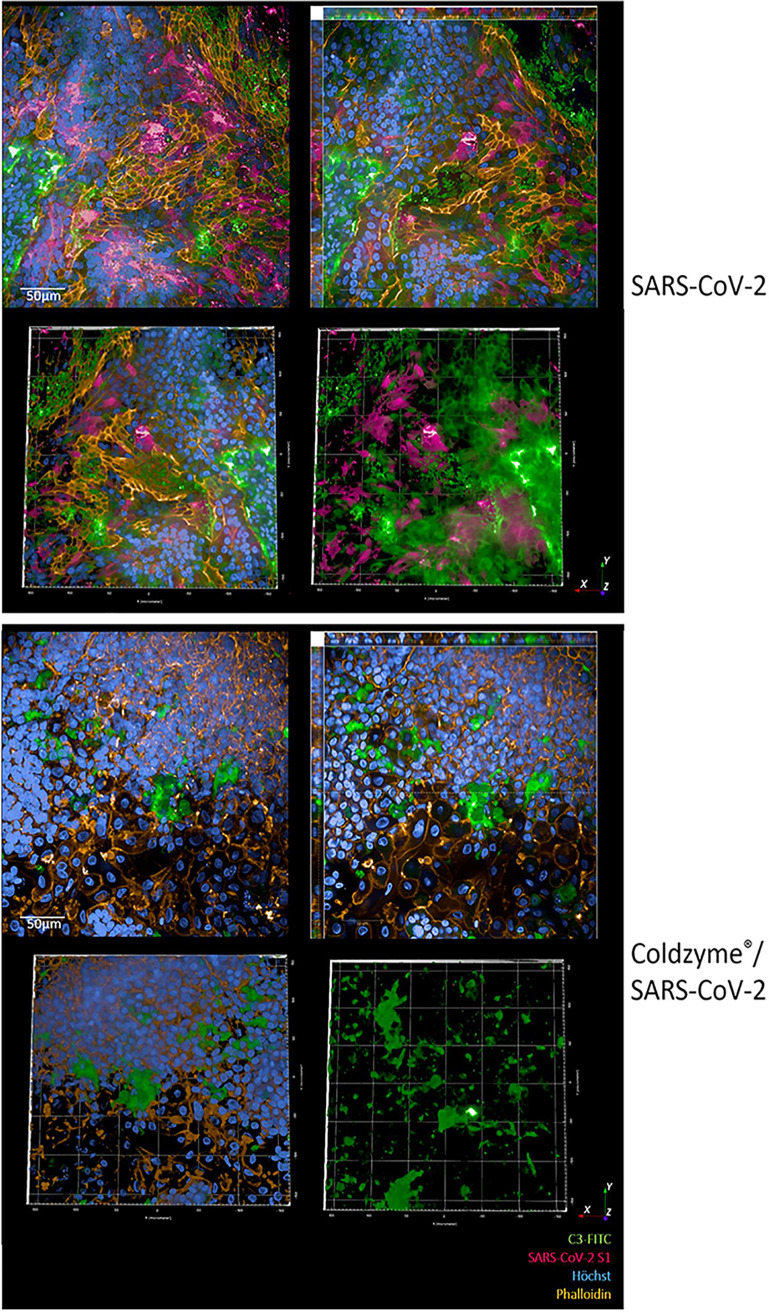
Increased infection of nasal epithelial cells using SARS-CoV-2 was impeded by pretreatment with ColdZyme. Visualization of SARS-CoV-2-infected pseudostratified nasal epithelia pretreated with ColdZyme mouth spray. Multilayered epithelia were pretreated for 30 min with ColdZyme sprayed apically and infected by apical addition of SARS-CoV-2 (MOI 0.1). d3pI cells were fixed and stained for Hoechst (blue), SARS-CoV-2-S1 (red), complement C3 (green), and phalloidin (orange) and then analyzed by HCS. The upper panel illustrates SARS-CoV-2-infected cultures, and the lower panel illustrates ColdZyme/SARS-CoV-2-infected cultures. Under each condition, one representative Z-stack (upper left), xyz stack (upper right), and 3D analyses of all stainings (lower left) and virus/C3 stainings (lower right) are depicted. Stainings were repeated thrice. Scale bars represent 50 μm.

Since SARS-CoV-2 entry factors are most highly expressed in nasal epithelial cells together with innate immune genes (21, 22), we analyzed whether the protective effects observed with ColdZyme mouth spray also apply to highly differentiated nasal epithelia. As illustrated in Fig. 3, these epithelia are highly permissive for SARS-CoV-2 infection (Fig. 3, SARS-CoV-2, red) accompanied by massive IC C3 mobilization (Fig. 3, SARS-CoV-2, green). Again, the virus and C3 signal were not only superficial as depicted by 3D and xyz analyses (Fig. 3, SARS-CoV-2, upper right and lower panel). Applying the ColdZyme mouth spray to these tissues was able to considerably decrease the viral load on d3pI, but nevertheless a high IC C3 induction and disrupted tissues were observable (Fig. 3, ColdZyme/SARS-CoV-2). These analyses revealed a protective mechanism against high SARS-CoV-2 infection observed in nasal epithelia by applying the ColdZyme mouth spray despite IC C3 mobilization. Due to the thinner layers of the nasal epithelia compared to HAE cultures, IC C3 mobilization and tissue damage could rely on the power of the spray appropriate for the oral cavity.

## DISCUSSION

Here, we found that one hub of ColdZyme mouth spray was sufficient to block SARS-CoV-2 from binding to and infecting highly differentiated, mucus-producing, and ciliated primary human nasal and bronchial airway epithelial tissue cultures.

Upon interaction of SARS-CoV-2 with pseudostratified epithelia, destruction of tissue culture integrity and a massive infection and intracellular C3 production were observed with concomitant anaphylatoxin C3a secretion. These effects were avoided by spraying ColdZyme onto human bronchial epithelial cells prior to infection with SARS-CoV-2. The protective effect from SARS-CoV-2 infection *in vitro* was observable up to 2 h following application of the ColdZyme mouth spray to the apical side of fully differentiated respiratory epithelia. A clinical trial on evaluation of ColdZyme in response to experimentally induced common cold applied 6 doses of the mouth spray daily, which corresponds to our time window of 1 to 2 h of effectiveness (clinical trial: COLDPREVII, https://clinicaltrials.gov/ct2/show/NCT02479750).

There is still a lack of therapeutic options against human coronaviruses such as SARS-CoV-2, HCoV-229E, or other common cold respiratory viruses (23). Coronaviruses are the causative pathogen for about one-third of common cold cases (23), and the worldwide SARS-CoV-2 pandemic has resulted in a global health crisis. Therefore, easy, fast, and already safety-tested measures to impede virus transmission from one individual to another are urgently needed. ACE2 and TMPRSS2, the entry factors for SARS-CoV-2, have been detected in multiple epithelial cells across the airway and in alveolar type II cells and are in particular highly expressed in nasal epithelial cells together with innate immune genes (21, 22). Further, the severity of COVID-19 has been associated with high viral load in the oral cavity (24). Elevated coexpression of SARS-CoV-2 host entry receptors with innate immune genes in nasal epithelia and upper respiratory tract might result in uncontrolled viral propagation and infection of lower airways. These two critical factors of infection have been identified to be elevated in patients with predisposing factors such as age, smoking, pollutants, temperature, and genetics (reviewed in reference 25). Since nasal and oral epithelia are portals for initial infection and transmission, in our study ColdZyme mouth spray was applied to normal human bronchial epithelial cells of the upper respiratory tract as well as primary nasal epithelial cells grown in air-liquid interphase. Comparable to studies of Vero E6 and MRC-5 monolayers on the efficacy of ColdZyme mouth spray, we here describe an efficient inactivation of SARS-CoV-2 by applying ColdZyme to both tissue cultures of human airway epithelial (HAE) cells. Binding of the virus was completely blocked, if HAE cells were pretreated with ColdZyme, which is probably due to its already described physical barrier function against common cold viruses. The mouth spray contains glycerol as well as trypsin from the Atlantic cod, which together form a protective barrier (8). Its efficiency in impeding virus binding to epithelial tissues can in part be explained by trypsin-mediated cleavage of arginine and lysine amino acid residues within the SARS-CoV-2 S protein as described by Gudmundsdottir et al. (8). Further, mucus production within the epithelia might be triggered due to applying the mouth spray. Protease inhibitors within the mucus membranes play an essential role in preventing penetration of negatively charged proteins, such as cod trypsin, through their glycosylated protein layers (26). This in turn could result in enhanced mucociliary clearance as also observed within our ColdZyme-treated epithelia, which showed an accumulation of fluorescently labeled SARS-CoV-2 antibodies only at the edges of the filters, when superficially imaging the whole filter. Such treated cultures did not show any virus deposition within the epithelial layers in contrast to heavily infected HAE epithelia without pretreatment using the ColdZyme mouth spray. Further, ColdZyme completely antagonized not only binding of SARS-CoV-2 but also infection and concomitant virus-induced tissue damage and local complement activation as indicators for innate immune activation. Exacerbation of injury by local complement activation in the airway epithelium was already shown for SARS-CoV infection (20, 26). Additionally, increased anaphylatoxin levels (C3a, C5a) in plasma and lung homogenates have been implicated in the pathogenesis of various lung conditions including cystic fibrosis and idiopathic pulmonary fibrosis (27, 28). Elevated anaphylatoxin levels resulted in downmodulation of regulators of complement activation such as CD55 and CD46 and changes in injury markers on HAE cells (27). As illustrated in our highly differentiated, pseudostratified 3D models, extremely high levels of C3a are secreted from the airway epithelium upon interaction with SARS-CoV-2 (20), which was significantly downmodulated by applying ColdZyme. Both C3a and C5a comprise important effector molecules attracting, activating, and regulating components of innate and adaptive immunity (29). Inflammation is among the first coordinated lines of defense following tissue damage by either infection or injury, but excessive immune responses convert the protective mechanism into a harmful one as observed in COVID-19 patients (30). Here, we demonstrated that local complement production and tissue damage—linked *in vivo* to poor outcome in COVID-19—were mediated by infection of HAE cells with SARS-CoV-2. These effects could be arrested in primary human nasal and airway epithelia by sole pretreatment of epithelia with ColdZyme. This resulted not only in the restoration of transepithelial electrical resistance, a marker of epithelial integrity, but also in significantly lowering viral loads, IC C3 production, and C3a secretion. Despite significantly decreasing SARS-CoV-2 viral loads in nasal epithelia, higher IC C3 production and tissue disruption were found by image analyses following ColdZyme treatment. These effects of increased IC C3 levels and tissue damage of nasal epithelia might be due to the power of the spray damaging the slightly thinner nasal epithelium (31). Although the nasal epithelial cells were at ALI for about 130 days, they, too, exhibited a thinner phenotype than 80-day-old NHBE cells.

In summary, we propose that the application of ColdZyme mouth spray represents a simple and promising approach in SARS-CoV-2 prevention or reduction in SARS-CoV-2-related symptoms by blocking virus binding and infection as well as concomitant extensive complement activation and tissue damage. Although the results from our realistic *in vitro* 3D models are not directly translatable into *in vivo* efficacy, they open up the exciting possibility that ColdZyme can be applied in the prevention of SARS-CoV-2 transmission and spread.

## MATERIALS AND METHODS

### Ethics statement.

Written informed consent was obtained from all donors of leftover nasopharyngeal/oropharyngeal specimens and EDTA blood by the participating clinics. The Ethics Committee of the Medical University of Innsbruck (a copy is attached to the proposal, ECS1166/2020) approved the use of anonymized leftover specimens of COVID-19 patients for scientific purposes.

### Cell culture of tissue models and ColdZyme treatment. (i) HAE.

Normal human bronchial epithelial (NHBE, Lonza, catalog no. CC-2540S) cells are available in our laboratory and routinely cultured in air-liquid interphase (ALI) as described previously (14, 15). Briefly, cells were cultured in a T75 flask for 2 to 4 days until they reached 80% confluence. The cells were trypsinized and seeded onto GrowDexT (UPM)-coated 0.33-cm^2^ porous (0.4-μm) polyester membrane inserts with a seeding density of 1 × 10^5^ cells per Transwell (Costar, Corning, NY, USA). The cells were grown to near-confluence in submerged culture for 2 to 3 days in specific epithelial cell growth medium according to the manufacturer’s instructions. Cultures were maintained in a humidified atmosphere with 5% CO_2_ at 37°C and then transferred to ALI culture. The epithelium was expanded and differentiated using airway medium from Stemcell. The number of days in development was designated relative to initiation of ALI culture, corresponding to day 0. MucilAir nasal cells were obtained from Epithelix-Sárl (Suisse), Geneva, Switzerland, and cultured according to the manufacturer’s protocol. One hub of ColdZyme mouth spray was applied to the apical side of the fully differentiated epithelia prior to infection using SARS-CoV-2. This corresponded to approximately 50 μl of liquid, evenly dispersed over the tissue culture. The apical application was carefully performed to not mechanically disrupt the epithelial surface.

### (ii) Vero/TMPRSS2.

VeroE6/TMPRSS2 is an engineered VeroE6 cell line expressing high levels of TMPRSS2 and highly susceptible to SARS-CoV-2 infection. This cell line was used to expand SARS-CoV-2 viruses from repositories as well as patient isolates. The cell line was obtained via the Center for AIDS Reagents (National Institute for Biological Standards and Control) and is described in the work of Matsuyama et al. (32).

### TEER measurement.

TEER values were measured using an EVOM voltohmmeter with STX-2 chopstick electrodes (World Precision Instruments, Stevenage, United Kingdom). Measurements on cells in ALI culture were taken immediately before the medium was exchanged. For measurements, 0.1 ml and 0.7 ml of medium were added to the apical and basolateral chambers, respectively. Cells were allowed to equilibrate before TEER was measured. TEER values reported were corrected for the resistance and surface area of the Transwell filters.

### Staining and high content screening (HCS).

To visualize SARS-CoV-2 infection in monolayers and 3D tissue models, cells were infected with clinical specimens of SARS-CoV-2 and analyzed for characteristic markers in binding experiments after 2 h or for infection experiments on day 3 postinfection (d3pI). After SARS-CoV-2 exposure, 3D cell cultures were fixed with 4% paraformaldehyde. Intracellular staining was performed using 1× intracellular staining permeabilization wash buffer (10×; BioLegend, San Diego, CA, USA). Antibodies to stain the cell surface (wheat germ agglutinin [WGA-680]; ThermoFisher Scientific, Waltham, MA, USA), nuclei (Hoechst 33342; Cell Signaling Technologies, Danvers, MA, USA), actin (phalloidin-Alexa 647; Cell Signaling Technologies, Danvers, MA, USA), and complement C3 (C3-fluorescein isothiocyanate [FITC]; Agilent Technologies, Santa Clara, CA, USA) were used. Intracellular SARS-CoV-2 was detected using Alexa 594-labeled SARS-CoV-2 antibodies against S1 and N (both from Sino Biological, Beijing, China). The Alexa 594-labeling kit was purchased from Abcam, Cambridge, United Kingdom. After staining, 3D cultures were mounted in Mowiol. To study these complex models using primary cells cultured in 3D and to generate detailed phenotypic fingerprints for deeper biological insights in a high-throughput manner, the Operetta CLS system (PerkinElmer, Waltham, MA, USA) was applied. Spot analyses and absolute quantification for SARS-CoV-2-containing cells (Harmony software) were performed on more than 1,200 cells per condition.

### Viruses.

Clinical specimens from COVID-19-positive swabs (ethics statement, ECS1166/2018) and SARS-CoV-2 viruses from repositories (BEI Resources, Manassas, VA, USA; CFAR/NIBSC; Nr-52281, Nr-52282, NR-52286) were propagated according to the manufacturer’s instructions and used subsequently to infect HAE tissue cultures.

### Profiling of anaphylatoxin C3a.

C3a secretion of HAE tissue models was detected by the BD OptEIA human C3a enzyme-linked immunosorbent assay (ELISA) kit (BD Biosciences, Franklin Lakes, NJ, USA) according to the manufacturer’s instructions.

### Statistical analysis.

Statistical analysis of differences in infection levels, TEER values, or cytokine production was performed utilizing the GraphPad Prism software and using one-way analysis of variance (ANOVA) with Tukey’s posttest.
